# Chemical-genetic profile analysis in yeast suggests that a previously uncharacterized open reading frame, *YBR261C*, affects protein synthesis

**DOI:** 10.1186/1471-2164-9-583

**Published:** 2008-12-03

**Authors:** Md Alamgir, Veronika Eroukova, Matthew Jessulat, Jianhua Xu, Ashkan Golshani

**Affiliations:** 1 Department of Biology, Carleton University, 1125 Colonel By Drive, Ottawa, K1S 5B6, Canada; 2 Ottawa Institute of Systems Biology, Carleton University, 1125 Colonel By Drive, Ottawa, K1S 5B6, Canada; 3 College of Life Science, JiLin University, Changchun, 130012, PR China

## Abstract

**Background:**

Functional genomics has received considerable attention in the post-genomic era, as it aims to identify function(s) for different genes. One way to study gene function is to investigate the alterations in the responses of deletion mutants to different stimuli. Here we investigate the genetic profile of yeast non-essential gene deletion array (yGDA, ~4700 strains) for increased sensitivity to paromomycin, which targets the process of protein synthesis.

**Results:**

As expected, our analysis indicated that the majority of deletion strains (134) with increased sensitivity to paromomycin, are involved in protein biosynthesis. The remaining strains can be divided into smaller functional categories: metabolism (45), cellular component biogenesis and organization (28), DNA maintenance (21), transport (20), others (38) and unknown (39). These may represent minor cellular target sites (side-effects) for paromomycin. They may also represent novel links to protein synthesis. One of these strains carries a deletion for a previously uncharacterized ORF, *YBR261C*, that we term *TAE1 *for Translation Associated Element 1. Our focused follow-up experiments indicated that deletion of *TAE1 *alters the ribosomal profile of the mutant cells. Also, gene deletion strain for *TAE1 *has defects in both translation efficiency and fidelity. Miniaturized synthetic genetic array analysis further indicates that *TAE1 *genetically interacts with 16 ribosomal protein genes. Phenotypic suppression analysis using *TAE1 *overexpression also links *TAE1 *to protein synthesis.

**Conclusion:**

We show that a previously uncharacterized ORF, *YBR261C*, affects the process of protein synthesis and reaffirm that large-scale genetic profile analysis can be a useful tool to study novel gene function(s).

## Background

The number of available sequenced genomes has provided the biologists with a wealth of sequence information containing thousands of genes. Many of these genes code for proteins with multiple functions, some of which are not known. Others code for proteins of completely unknown function(s). To tackle this challenge, several large-scale methodologies, under the term functional genomics, have been developed which aim at revealing putative gene functions [1-3]. Due to its simple genetics, ease of manipulation, and conserved pathways, the yeast *Saccharomyces cerevisiae*, emerged as a model organism of choice for functional genomics [4]. While significant knowledge has been gained from various large-scale investigations, more experiments are needed to uncover the details of the functions of genes involved in different cellular processes. Exploring the function of individual proteins can greatly advance our understanding of the biology of a cell as a system.

Genes, which are involved in similar pathways often genetically, interact with each other. Therefore, one way to study gene functions is to investigate the interactions they make with each other [3]. This is based on the assumption that many eukaryotic pathways are functionally redundant. Thus, deletion of a gene may be tolerated with no phenotypic consequences. Inactivation of a second functionally related gene however, can cause sickness or even lethality. Therefore sickness of double mutants or "synthetic lethality" has been used to reveal novel gene functions. In simple terms, synthetic genetic array (SGA) analysis refers to large-scale investigation aimed at examining gene functions using double gene knockouts [3].

In addition to its role in functional genomics, availability of the yeast non-essential gene deletion array (yGDA, approximately 4700 strains) also provided the opportunity to investigate the cellular target sites of inhibitory compounds [5-7]. In this way, compounds with unknown cellular target sites are examined for their inhibitory effects on yGDA. The hypersensitive strains for genes with known functions are used to form a genetic profile for the activity of the target compound. This provides a fast and effective way to investigate cellular target sites of inhibitory compounds.

Similarly, inhibitory compounds with known modes of activity could be used to detect novel gene functions. This is not a novel concept and in various small-scale studies, numerous gene functions have been examined based on the increased sensitivity of their gene deletion strains to different compounds [8,9].

As a final step in the gene expression pathway, the regulation of protein synthesis (translation) is used to control the expression of a variety of genes under different physiological conditions. For example, during cell division in the early steps of embryonic development [10,11], or during cellular transformation and cancer development [12], and as well, in stress conditions and apoptosis [13].

Even though the underlying principles of translation machinery have been the subject of vigorous investigations over the last few decades, details of all translation related proteins, protein complexes and pathways, as well as their communications and cross-talks with other cellular processes, have not been fully elucidated. Recently, several large-scale genomic investigations have uncovered numerous novel proteins thought to be functionally related to protein synthesis in *S. cerevisiae *[14-16], suggesting that there remain other undiscovered translation proteins.

Here, we applied a large-scale chemical-genetic profile analysis to identify yeast deletion strains that show increased sensitivity to aminoglycoside antibiotic paromomycin. This compound exerts its activity by targeting the process of protein synthesis. Focused follow-up experiments provided evidence that *YBR261C*, a previously uncharacterized open reading frame (ORF) which is identified by this screen, affects the process of protein synthesis in yeast.

## Results

### The Initial screening and identification of TAE1

In order to identify genes that affect protein synthesis, we screened the entire set of yGDA (~4700) for increased sensitivity to the aminoglycoside paromomycin. Paromomycin binds to the small ribosomal subunit of eukaryotic cells and compromises the translation fidelity [17]. Previously, it was shown that deletion of certain translation related genes caused increased sensitivity to paromomycin [18,19]. Therefore, we employed hypersensitivity to this drug as a way to detect novel gene candidates involved in translation. It should be noted that the deletion of certain translation related genes would cause increased resistance to paromomycin. This however, has not been investigated in our analysis.

We used yeast colony size reduction (CSR) as a tool to detect sensitivity to drug treatments. We have previously shown, that based on the parameters used by us (see Materials and Methods), CSR analysis can detect approximately 63% of the sensitive strains that are detected by a large-scale spot test (ST) analysis with no repeats [20]. Hence, there are a number of sensitive strains that would be missed by our analysis, that might otherwise be detected by ST. Similarly, the same large-scale ST analysis failed to detect 59% of the strains detected by CSR, which may represent novel/false-positives associated with CSR.

Here, we use sensitivity to paromomycin as a tool to detect protein synthesis related genes. A draw back for this, as well as other similar drug-based screening tests, is the detection of sensitive strains for those genes with no direct relation to the activity of the target drugs. A major source of such false-positives in our experiments may stem from those genes that play a role in general stress conditions. Multi-drug resistant genes represent some of these examples. For instance, it has been shown that the deletion of *QDR1*, a transporter gene and a member of efflux pumps, confers sensitivity to several unrelated drugs [21]. To increase the specificity of our selection procedure, we coupled our initial screen with a secondary search (based on the same parameters) for increased sensitivity to a second drug, which has no reported activity on the process of translation. For this purpose, we selected calcofluor white (CW), which is known to inhibit cell wall function by binding with chitin [22]. In this way, only those gene deletions that conferred sensitivity to paromomycin alone may represent meaningful positives.

**Figure 1:**
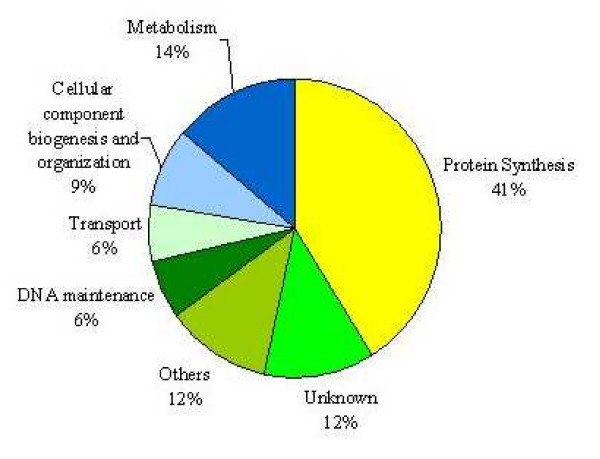
**Categories of gene deletion strains with increased sensitivity to paromomycin**. Distribution of paromomycin hypersensitive yeast deletion strains in percentages according to their cellular functions.

As expected, our large-scale approach identified numerous translation genes such as *TEF4 *(translation elongation factor EF-1 gamma), *HCR1 *(a component of translation initiation factor 3), *RPS18B *(a ribosomal protein of small subunit), etc, which are sensitive to paromomycin and not to CW. The complete list of these genes is found in Additional file 1. The list of the genes that were sensitive to both paromomycin and CW is found in Additional file 2. Of the 325 gene deletions sensitive to paromomycin alone, we found 42 genes that often appeared in our similar drug screenings using different bioactive compounds. These may represent false-positives and should be treated with caution. From the 325 total reported genes, 134 have been previously linked to the overall process of protein biosynthesis. 191 of them however, have never been connected in any way to this process, and therefore, may also represent novel/false-positive genes. These genes can be further classified into 5 smaller categories based on the cellular processes in which they participate, plus those which are unknown. As indicated in Figure 1, the minor categories are: metabolism with 45 genes, cellular component biogenesis and organization with 28, DNA maintenance with 21, transport with 20 and others with 38 genes. There were also 39 genes, which are unknown. An explanation for these smaller categories is that they may represent minor cellular target sites (side-effects) for paromomycin. Alternatively, they may simply represent false-positives (see above). It is also possible that some of these genes might have novel roles during translation. In this case, they may represent communication bridges between different cellular processes and protein synthesis.

**Figure 2:**
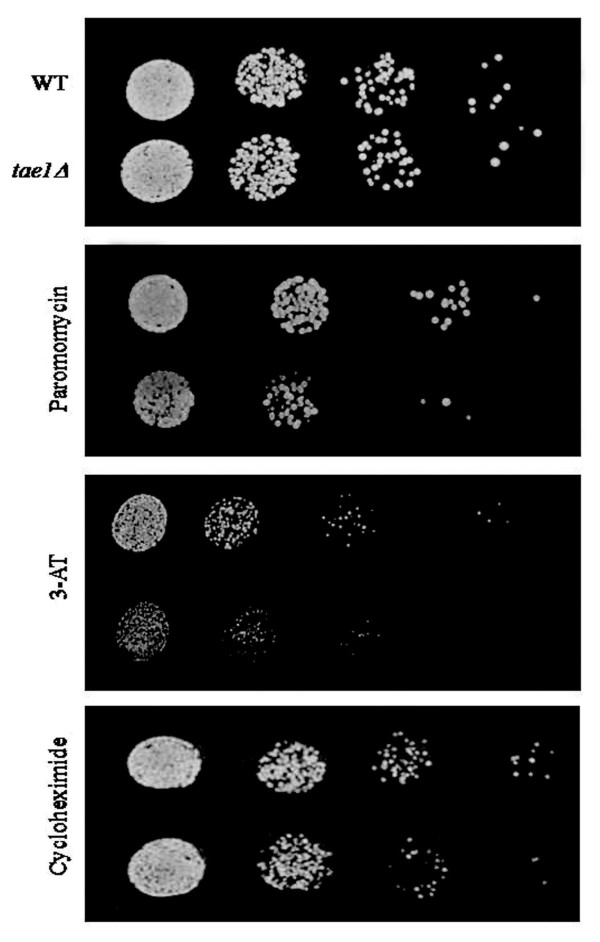
**Increased sensitivity of *tae1*Δ to different translation inhibitory drugs**. Deletion of *TAE1 *confers increased sensitivity to different drugs that target translation. Decreasing numbers of wild type and mutant (*tae1*Δ) yeast cells were spotted on solid media. The media was supplemented with sub-inhibitory concentrations of paromomycin (13 mg/ml), 3-AT (22 mg/ml), cycloheximide (45 ng/ml), or none (used as a control and shown in the top panel). Standard organic (YEPD) media was used for cycloheximide and synthetic complete (SC) media was used for paromomycin and 3-AT plates, and for the control plate shown here. Yeast cells were grown to mid-log phase and diluted 10-3 to 10-6 folds. Twenty microliters of each dilution (gradually decreasing) was spotted onto the media and grown at 30°C for 1–2 days. Deletion of *TAE1 *conferred increased sensitivity to paromomycin and 3-AT. Occasional sensitivity to cycloheximide was assumed to be an artifact.

One of the previously uncharacterized ORF identified in this screen is *YBR261C*. There is no reported information about this ORF, except that it is computationally predicted to have a methyltransferases domain [23]. We therefore hypothesized that this might be a novel translation related gene. We termed this ORF, *TAE1*, for Translation Associated Element 1, and subjected it to further analysis for its potential involvement in protein synthesis. ST analysis was used to confirm our large-scale observations (Figure 2). When the growth media was supplemented with a sub-inhibitory concentration of paromomycin (13 mg/ml), a deletion strain for *TAE1 *(*tae1*Δ) showed a reduction in its growth pattern (Figure 2).

We then examined *tae1*Δ strain for its increased sensitivity to 3-amino-1,2,4-triazole (3-AT) and cycloheximide. 3-AT can affect translation by altering the pool of amino acids in the cell [24]. Cycloheximide binds to large ribosomal subunit [25], and inhibits translation elongation by interfering with tRNA translocation [26]. It has previously been reported that deletion of certain genes that affect translation may confer sensitivity to multiple drugs that target translation. For example, deletion of Sfp1, which regulates ribosome biogenesis, confers increased sensitivity to both cycloheximide and paromomycin [27]. Shown in Figure 2, our spot test analysis indicated that in addition to paromomycin, *tae1*Δ also showed increased sensitivity to 3-AT. Also some sensitivity for *tae1*Δ to cycloheximide was occasionally observed. However, due to irreproducibility of these observations, this sensitivity was presumed to be an artifact.

During the preparation of this manuscript, a new study was reported in which sensitivity of yeast gene deletion mutants to paromomycin were investigated using a heterozygous diploid yeast gene deletion mutant array [28]. This system is different from ours (haploid based), as its diploid mutant strains always carry a copy of the wild type genes and consequently show less obvious growth defects [29]. Of the 51 mutant strains that were identified by the authors as sensitive to paromomycin, 39 are shared between the two systems. Of these, 16 mutants were also identified by our haploid system. It is worth mentioning that *tae1*Δ was not detected to have increased sensitivity to paromomycin in the heterozygous system, further highlighting the difference between the two systems [28].

### The effect of *TAE1 *gene deletion on protein synthesis

Translation genes can be involved in different aspects of translation. To examine the involvement of novel genes in protein synthesis, we divided translation into three general categories. Group one includes those genes that are associated with ribosome biogenesis, group two contains genes that alter translation efficiency, and group three is composed of the genes that affect translation fidelity. Depending on their molecular function(s), some translation genes may fall into none [30], one [27,31] or more [32] of these three categories. If *TAE1 *is involved in the process of translation, it might fall into one or more of these categories.

### a) Involvement of TAE1 in ribosome biogenesis

**Figure 3:**
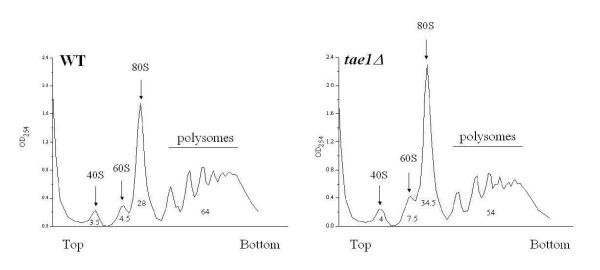
**Ribosome profile analysis of yeast strains**. Deletion of *TAE1 *results in an overall decrease in polysomes, and a corresponding increase in 80S monosome formations. There is also an increase in 60S subunit in *tae1*Δ cells. The large polysomes were collected at the lower half of the gradients (x-axis). The values under the peaks represent the areas under the curve for each peak, in percentages. Each experiment was repeated three times with similar outcomes.

Ribosome biogenesis and assembly is one of the most important processes in the protein synthesis pathway [33]. This process might be explained as the overall step that leads to the formation and assembly of functional ribosomes and includes regulation of pre-rRNA transcription, and its corresponding mechanisms, pre-rRNA processing, rRNA transport, rRNA maturation, ribosome assembly, etc. [34]. We therefore reasoned that depending on the molecular function(s) of *TAE1*, if this gene is involved in ribosome biogenesis or assembly, its deletion might result in the alteration of the profile of ribosomal subunits. To examine this possibility, ribosome profile analysis was performed. As expected, distinct 40S and 60S subunit peaks, as well as 80S monosomes and polysome peaks (Figure 3) were detected in both the wild type and *tae1Δ *strains. In addition, *tae1Δ *strain showed a reduction in polysomes and corresponding increases in 80S monosomes and 60S subunits (Figure 3). These alterations in ribosomal profile further indicate the involvement of *TAE1 *in protein synthesis.

### b) Involvement of TAE1 in translation efficiency

**Figure 4:**
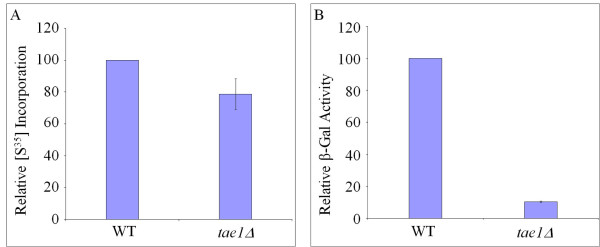
**Protein synthesis profile in the presence and absence of Tae1**. In (A) total protein synthesis is measured using [^35^S] methionine incorporation. The average count for [^35^S] methionine incorporation for wild type is 11,356,073 (± 1,300,000) counts, which is set to 100%. In the absence of Tae1 protein, total protein production is reduced by approximately 22%. t-test analysis indicates that the observed difference is statistically significant with the p-value of 0.05. In (B) the efficiency of protein synthesis is measured using an inducible β-galactosidase reporter gene. The average β-galactosidase activity for wild type is 7.51 (± 0.6) units, which is set to 100%. In the absence of Tae1 protein, an approximately ten-fold reduction in β-galactosidase production is observed.

If *TAE1 *is involved in protein synthesis, then based on its molecular function(s), its deletion may alter the cell's efficiency s to synthesize proteins. To investigate this possibility, we used [^35^S] methionine incorporations to measure the rate of total protein synthesis in different strains. As indicated in Figure 4A, it was observed that on average, *tae1Δ *had a reduced level of [^35^S] methionine incorporation (approximately 22%) compared to the control strain. To confirm this observation, we used an inducible β-galactosidase reporter construct (Figure 4B). It was observed that the deletion of *TAE1 *reduced the level of β-galactosidase synthesis from an inducible expression plasmid by approximately ten-folds (Figure 4B). These observations suggest that deletion of *TAE1 *may reduce the efficiency of protein synthesis in a cell, and provide further support that *TAE1 *affects protein synthesis.

### c) TAE1 and Translation fidelity

**Figure 5:**
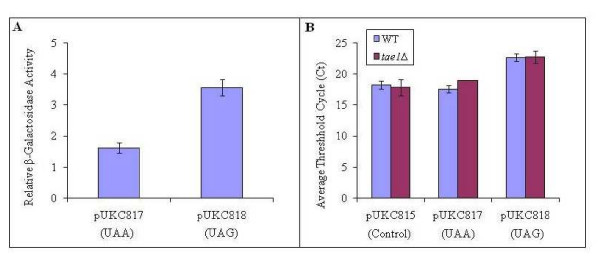
**Effect of *TAE1 *deletion on translation fidelity**. (A) Deletion of *TAE1 *resulted in increased levels of β-gal activity from the *lacZ *reporters, pUKC817 and pUKC818, containing premature stop codons, UAA and UAG, respectively. The relative β-gal activity is determined by normalizing the activity of the mutant constructs (pUKC817 and pUKC818) to the control construct (pUKC815), and related to that obtained by the wild type strain (for example, the value for pUKC817/pUKC815 in *tae1*Δ cells is related to that of pUKC817/pUKC815 in the wild type cells). The average β-gal activity for the wild type strain transformed with pUKC815 is 19.05 (± 1.1) units. (B) Q-RT-PCR analysis indicates that alterations for the relative contents of LacZ mRNAs do not explain the difference in β-gal activities observed in (A). The C_t _for the control and experimental samples were calculated from the threshold cycles. pUKC815 is the background construct without a premature stop codon and used as a control.

To make a functional protein, the fidelity of protein synthesis is maintained at almost all stages of translation. This fidelity is controlled during the start site selection and elongation when the mis-incorporation of the wrong amino acid may alter the integrity of the final product, as well as during termination, when a stop codon might be read through. If *TAE1 *is involved in translation, then it might be expected that based on its molecular function(s), the deletion of this gene may alter the fidelity of translation. Translation fidelity can be studied using specialized expression systems such as those that contain β-galactosidase expression cassettes, with premature stop codons [35]. In this investigation, we used plasmids pUKC817 and pUKC818 that contain *lacZ *genes carrying the nonsense codons UAA and UAG, respectively [35]. It was observed that the deletion of *TAE1 *resulted in an increased level of read through for the nonsense codons investigated, suggesting that in this deletion strain, the translation fidelity seem to be compromised (Figure 5A). For unknown reasons, the average values for β-galactosidase activities in the wild type strain, were systematically higher than expected.

Since differential levels of β-galactosidase activity in above experiments may also stem from altered levels of mRNAs, the content of β-galactosidase mRNAs of WT and *tae1*Δ were investigated using Q-RT-PCR. We observed no significant variation in the amounts of these mRNAs (Figure 5B), which could explain the observed difference for the β-galactosidase activities. It was therefore concluded that the observed differences likely stem from translation read through. Altogether, the results indicate that the deletion of *TAE1 *seems to compromise translation fidelity, providing further evidence that *TAE1 *affects translation.

### TAE1 genetically interacts with translation related genes

**Figure 6:**
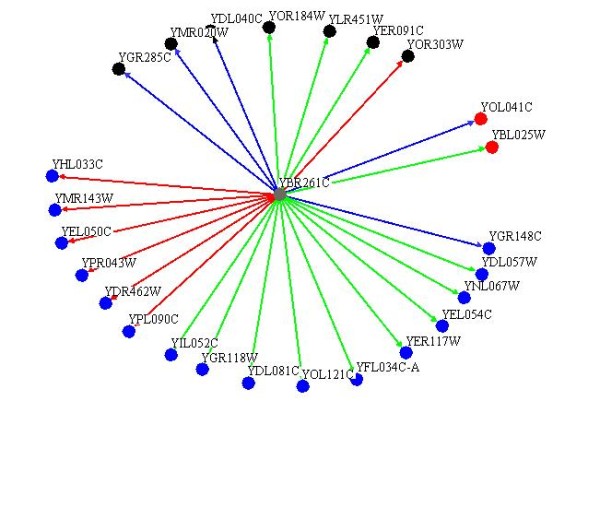
**Genetic interaction of *TAE1 *with translation genes**. *TAE1 *genetically interacts with numerous translations related genes to produce sick phenotypes. These interacting genes could be further divided into two major categories of ribosomal subunits (16 genes, blue circles), and amino acid and protein production (7 genes, black circles). The interacting genes shown in red circles are involved in ribosome biogenesis. Strong, moderate and weak interactions are shown by red, green and blue lines, respectively.

The genes that are functionally related and are involved in similar pathways, often genetically interact with each other. Consequently, studying the genetic interactions of a novel gene is often used as a method to infer the function of that gene [36,37]. If *TAE1 *is a true translation gene, then it might be expected that *TAE1 *would genetically interact with certain known translation associated genes. To investigate this possibility, we examined the genetic interactions of *TAE1 *with a set of 384 genes, which are known or thought to be involved in translation. As indicated in Figure 6, it was observed that *TAE1 *genetically interacted with numerous translation related genes to produce sick phenotypes, which are classified as i) very sick, ii) sick, and iii) moderate. Lethal interactions were not considered. The interactions between *TAE1 *and translation related genes were further divided into three categories with ribosomal proteins forming the largest cluster (16 genes), followed by those involved in amino acids and protein production (7 genes), and those involved in rRNA synthesis (2 genes). Descriptions of these genes are listed in Additional file 3. The fact that *TAE1 *genetically interacts with different translation associated genes provides further evidence for the involvement of *TAE1 *in the process of translation.

### Phenotypic suppression by the overexpression of TAE1

To examine the cellular activity of Tae1 protein, we employed a high throughput approach, based on the phenotypic suppression of the gene deletion mutants that have known functions. Deletion of genes, which are involved in a specific pathway, may cause increased sensitivity to treatments that target the same process. Such hypersensitivities can be compensated by the overexpression of other genes with similar cellular functions. For example, it has previously been reported that the absence of Yku80, involved in telomere maintenance, causes increased sensitivity to elevated temperature. Overexpression of either Est2, a catalytic subunit of telomerase, or Tlc1, the RNA template component of telomerase, compensated for the absence of Yku80 and reversed the heat hypersensitivity of *yku80Δ *[38].

**Figure 7:**
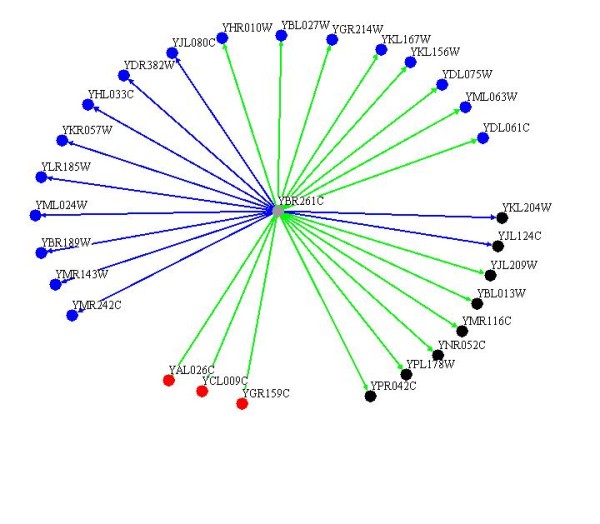
**Overexpression of *TAE1 *phenotypically suppresses the hypersensitivity of numerous translation genes against drug treatments**. Overexpression of *TAE1 *suppressed the inhibitory effects of neomycin and/or streptomycin for 28 yeast gene deletion strains. Among them, 17 genes belong to ribosomal proteins (blue circles) and 8 genes are involved in translation control (black circles). Red circles represent other genes. Complete and partial suppressions are shown by green and blue lines, respectively.

Here, we investigated the activity of Tae1 protein by examining the effect of its overexpression in suppressing hypersensitivities to antibiotics neomycin and streptomycin for the above 384 translation related gene deletion yeast strains. Like paromomycin, the antibiotics neomycin and streptomycin, belong to the aminoglycoside family, which binds to ribosomes and disrupts translation [30]. We observed that overexpression of Tae1 protein, suppressed the drug sensitivity phenotypes for 28 deletion mutants of known translation genes (Figure 7). These 28 mutants showed sensitivity to treatment with neomycin and/or streptomycin. Tae1 overexpression however, reversed the observed drug sensitivities. These 28 gene deletion mutants can be categorized into two main groups of gene deletions for ribosomal proteins (17 genes), and those for translation control proteins (8 genes). Descriptions of the deleted genes are listed in Additional file 4. The fact that overexpression of Tae1 suppresses the phenotypes of deletion mutants for translation genes, further confirms an involvement for *TAE1 *in protein synthesis.

## Discussion

Annotating gene functions has been an important aspect of post-genomic era. Identifying "which gene does what" is one of the fundamental tasks of systems biology, and sets the basis for understanding the biology of a cell. There are currently numerous uncharacterized genes with no known functions [4]. Moreover, there are numerous genes with multiple functions, some of which are not yet elucidated.

One approach to study gene function is to make gene knockouts, and observe the mutant cells' behavior to internal and/or external stimuli. Here, we screened the set of yGDA for sensitivity to paromomycin, which is known to target protein synthesis machinery. Due to an increased sensitivity, we hypothesized that a yeast gene deletion strain for *TAE1 *might affect the process of protein synthesis. We observed that the deletion of *TAE1 *reduced translation fidelity. This observation might be expected, since paromomycin is known to decrease the fidelity of translation. We also observed that deletion of *TAE1 *reduced the efficiency of translation, which is not necessarily coupled with translation fidelity, suggesting a wide-range effect for *TAE1 *on translation.

Tae1 protein is found in the cytoplasm and is bioinformatically predicted to contain an S-adenosylmethionine-dependent methyltransferase activity. Certain members of this family of proteins have been shown to methylate different components of the translation machinery. For example, DIM1 and SPB1, which are nucleolar proteins involved in rRNA methylation; TRM proteins (such as TRM1 and TRM2), which are tRNA methyltransferases found in both cytoplasm and nucleus; and Mtq2, which methylates translation release factor SUP45 and is found in both cytoplasm and nucleus. Presuming that Tae1 has a methyltransferase activity, we can assume that Tae1 affects translation by methylating a component of translation machinery.

In agreement with the observed reduction in translation efficiency for *tae1Δ*, our ribosomal profile analysis indicated an overall decrease in polysomes when *TAE1 *was deleted. In contrast, the 60S free subunits were specifically accumulated in *tae1Δ *cells. Since 40S and 60S subunits are in equilibrium with 80S monosomes, the increase in 60S subunit may indicate a defect in 40S biogenesis [39]. Assuming that *TAE1 *is a methyltransferase, a possible explanation is that *TAE1 *may effect 40S biogenesis by either methylating 18S rRNA directly, or by methylating a factor which affects 40S biogenesis. In agreement with a role for *TAE1 *in ribosome biogenesis, *TAE1 *is found to be co-regulated with a number of ribosome processing factors in at least five different microarray analyses [40].

There is also an accumulation of 80S monosomes in *tae1Δ *cells, which may indicate a defect in translation initiation. Formation of defective ribosomes in the absence of *TAE1 *that cannot readily start elongation, may explain this accumulation of 80S monosomes. Alternatively, it is possible that *TAE1 *might affect translation initiation by modifying a translation initiation protein. The latter explanation however, cannot explain the accumulation of 60S subunits. Regardless, further experiments are required to investigate details of the molecular activity of *TAE1*, and to identify its potential substrate(s).

Our genetic analysis revealed that *TAE1 *genetically interacts with a number of translation genes. In accord with the above-suggested function for *TAE1 *in ribosome subunit biogenesis, the majority of the observed interactions were found to be with ribosomal protein genes. Similarly, our phenotypic suppression analysis indicated a functional compensation by *TAE1 *overexpression, for the absence of 17 different ribosomal proteins against drug treatments. It should be noted that our genetic and phenotypic suppression analysis resulted in two different sets of proteins. This is expected, as genetic interaction analyses generally target the genes involved in different pathways within a process (redundant pathways), whereas phenotypic suppression analyses generally target genes within the same pathway. This data can be further used to study the detailed mechanism of *TAE1 *activity.

## Conclusion

In conclusion, we investigated the sensitivity of yGDA to paromomycin, a drug that targets the process of translation. One of the mutant strains identified by our screen was a deletion strain for a previously uncharacterized ORF, *YBR261C*, that here we termed *TAE1*, for Translation Associated Element 1. Our follow-up experiments indicated that the deletion of *TAE1 *caused reduction in translation efficiency and fidelity. Deletion mutant strain for *TAE1 *also had an altered ribosome profile. Our genetic analyses further confirmed the involvement of this gene in translation. Identification of a new gene in the process of translation suggests that there may exist other novel translation genes, which are yet to be discovered.

In addition, the large-scale phenotypic suppression analysis used here can set the path for similar approaches to investigate other gene functions. Furthermore, our data also reaffirms that large-scale chemical-genetic profile analysis can be successfully used in functional genomics.

## Materials and methods

### Drug resistance screening for yeast gene deletion array

Sub-inhibitory concentrations of drugs were estimated from minimum inhibitory concentration (MIC) measurements based on a 96 varied concentration format as described in [41].

Approximately 4700 *MAT***a **haploid yeast, *S. cerevisiae *strains (BY4741, *MATa ura3Δ0 leu2Δ0 his3Δ1 met15Δ0*) from the non-essential Gene Deletion Array (yGDA) described in [3,5] was manually arrayed onto agar plates as previously described [36] in a 384 array format, with sub-inhibitory concentrations of paromomycin (13 mg/ml), CW (40 μg/ml) or without a drug (control). All plates were incubated at 30°C for 1–2 days. The sensitivity of the gene deletion array to different drugs was investigated as before [36]. In brief, different strains are pinned on two plates, one with a sub-inhibitory concentration of a target drug and one without (used as a control) and incubated at 30°C for 1–2 days. Digital images of plates were used to analyze the growth of individual colonies. For every plate the average size of colonies (white pixels) was calculated from equation (1).

(1) $$S_{ave}=1/N\sum_{i=1}^{N}S_{i}$$

where *N *is the total number of colonies present in a given plate and *S*_*i *_is the area of object *i*. The deviation of area for each colony from the plate's average area was used for further analysis (relative growth) and was calculated by subtracting the scalar *S*_*ave *_from the plate's ordered area array explained in equation (2).

(2) *ΔS*_*i *_= *S*_*i *_- *S*_*ave*_; *i *= 1, ..., 384 (16 × 24 = 384)

Relative colony size reduction of more than 30% was counted as a "hit". Each experiment was repeated three times. The gene deletion strains, which were "hits" in two or more of the three experiments, were counted as positives. Colonies with the highest two average reductions of 61% or more and 30–60% were defined supersensitives and sensitives, respectively.

ST test analysis was performed by growing yeast cell cultures in YEPD media to mid-log, following 10-3 to 10-6 folds dilutions. Twenty microliters of each dilution (gradually decreasing), was then spotted onto media containing the sub-inhibitory concentrations of the drugs (13 mg/ml paromomycin, 22 mg/ml 3-AT, and 45 ng/ml cycloheximide), and without (control). YEPD media was used for cycloheximide and SC media was used for paromomycin and 3-AT plates. The growth patterns were compared after 1–2 days at 30°C as in [42].

### Gene expression analysis

Constitutively expressed β-galactosidase (using pUKC815, pUKC817 and pUKC818) was assayed as described [43]. The units of enzyme activity were calculated as nanomoles of O-nitrophenyl-α-D-galactopyranoside (OPNG) hydrolyzed per microgram of total protein [44]. All assays were conducted in triplicate. Induced β-galactosidase (using p416) was assayed as before [45].

*In vivo *[^35^S] methionine incorporation was performed as previously described by Schwartz and Parker [46] with modifications. Briefly, yeast strains were grown to mid-log phase at 30°C in YEPD. The cells were harvested, resuspended in pre-warmed minimal medium lacking methionine, and supplemented with 10 μCi/ml of [^35^S] methionine. The cells were incubated for 1 h at 30°C and harvested by centrifugation. The samples were then washed with distilled water six times and collected (1 μl aliquot) on Whatman paper. The paper was air dried and exposed to storage phosphor screen for 1 h. The counts were normalized to the final cell totals. Each experiment was repeated at least four times.

Total RNA was isolated using Bio-Rad RNA isolation kit. cDNA was constructed from 0.5 μg of total RNA of each strain using iScript cDNA synthesis kit with SYBR green supermix (Bio-Rad) according to the instructions of the manufacturer. The quantification of mRNA was performed using real-time RT-PCR (Q-RT-PCR) on a Rotor-Gene RG-300 from Corbett research. The PCR quantification and melting curves were generated using the Rotor gene 6 software. The amplification was performed: initial denaturation 95°C for 10 min followed by 40 cycles at 95°C for 30 s, 55°C for 20 s and 72°C for 20 s each with using 50 nmoles each of the forward (5'-ACTATCCCGACCGCCTTACT) and reverse (5'-TAGCGGCTGATGTTGAACTG) primers. The fluorescence was read at the end of each round of amplification. All standard dilutions and samples were run in triplicate. Quantification of mRNA were achieved by comparing the threshold cycle (*C*_t_) value of the sample RNA from deletion strain with the *C*_t _value of WT strain's standard RNA [47].

### Ribosome profile analysis

Ribosome profiling was performed as described by Foani *et al *[48] with the following modifications. Wild type and mutant yeast cells were grown in YEPD at 30°C to a density of 2 × 10^7 ^cells/ml. Cycloheximide (50 μg/ml) was added to each culture and the cultures were quickly chilled in ice water bath. Cells were then centrifuged at 4000 rpm for 4 min at 4°C by sorvall SLA-1500 rotor. Cell pellets were resuspended in 10 ml of ice-cold YA buffer (breaking buffer A: 10 mM Tris-HCl [pH 7.4], 100 mM NaCl, 30 mM MgCl_2_, cycloheximide 50 μg/ml, heparin 200 μg/ml) and centrifuged 4000 rpm, 4 min, at 4°C (Sorvall SS34 rotor), twice. The pellets were then resuspended in 0.5 ml of YA buffer by vortex and stored at -80°C till the next day. Cells were then thawed on ice water bath. Glass beads were added and vortex for 20 sec at maximum speed, 10 times with 30 sec interval. The supernatants were centrifuged at 8,000 × g and 10,000 × g for 10 min and 30 min, respectively. Twenty OD_260 _units of each supernatant were fractionated on 8–48% sucrose gradients containing 50 mM Tris-acetate (pH 7.0), 50 mM NH_4_Cl, 12 mM MgCl_2_, and 1 mM dithiothreitol. The extracts were then centrifuged for 2.5 hrs at 39,000 rpm using a SW40-Ti rotor in a Beckman LE-80K at 4°C. The ribosome profiles were analyzed from the collected gradient solutions by monitoring the absorbance at 254 nm. Each experiment was repeated three times with similar results.

### Genetic interaction and phenotypic suppression analysis

Genetic interactions between *TAE1 *and a set of yeast gene deletion strains for 384 genes known or thought to be involved in translation were assessed by synthetic genetic miniarray profiling as discussed in [49] and the colony growth differences were assessed as in [20]. Possible synthetic sick interactions were confirmed by a spotting assay explained in [50]. We divided these interactions into strong, moderate and weak interactions based on their double mutant phenotypes of very sick, sick and slightly sick.

The *TAE1 *overexpression construct, p*GAL1/10*-GST-6xHis-*YBR261C *is obtained from the yeast gene overexpression array explained in [51]. A compatible *MATα *strain (Y7092, *MATα can1Δ::STE2pr-Sp_his5lyp1Δhis3Δ1 leu2Δ0 ura3Δ0 met15Δ0*) is then transformed with this construct and was crossed with a set of yeast gene deletion strains for 384 translation genes (see above) as in [49]. Sensitivity of yeast strains with or without *TAE1 *overexpression, against neomycin and streptomycin was performed using colony size measurements as discussed in [20]. Phenotypic complementations were divided into two categories of complete and partial suppressions. Partial complementation against both drugs is assigned partial. Complete complementation against one drug, regardless of the other, is assigned complete.

## Abbreviations

yGDA: yeast non-essential Gene Deletion Array; ORF: open reading frame; Tae1: Translation Associated Element 1; 3-AT: 3-Amino-1, 2, 4-triazole.

## Authors' contributions

All authors contributed to the conceptual development of the project. MA and VE conducted the experiments and analyzed the results. JX and AG administered the experiments. MA, MJ and AG wrote the manuscript. All authors read and approved the final manuscript.

## Supplementary Material

Additional file 1 ***Supplemental Table 1*.** List of genes deletions, which are sensitive to paromomycin. Click here for file

Additional file 2 ***Supplemental Table 2*.** List of genes deletions, which are sensitive to both paromomycin and calcofluor white (CW). Click here for file

Additional file 3 ***Supplemental Table 3*.** Descriptions of translation related genes that genetically interact with *TAE1*. Click here for file

Additional file 4 ***Supplemental Table 4*.** Descriptions of translation related genes that are phenotypically suppressed by overexpression of *TAE1*, against treatment with neomycin and/or streptomycin. Click here for file
