# Supplementary material for: Chemical-genetic profile analysis in yeast suggests that a previously uncharacterized open reading frame, YBR261C, affects protein synthesis
Source: BMC Genomics. 2008 Dec 3;9:583. doi: 10.1186/1471-2164-9-583 (PMC2613417; doi:10.1186/1471-2164-9-583)
Supplement: Additional file 3 — Supplemental Table 3. Descriptions of translation related genes that genetically interact with TAE1. [file 1471-2164-9-583-S3.doc]

Supplemental Table 3: Descriptions of translation related genes that genetically interact with *TAE1*

| Standard Gene Name | Systematic Gene Name | Phenotype | Description and Cellular Function |
| --- | --- | --- | --- |
| Ribosomal Proteins | | | |
| YDL075W | RPL31A RPL34 | Sick | Ribosomal protein component of the large (60S) subunit, similar to Rpl31Bp and rat L31 ribosomal protein |
| YDL081C | RPP1A RPLA1 | Sick | Ribosomal stalk protein involved in the interaction between ribosome and translational elongation factors |
| YDR462W | MRPL28 | Very sick | Mitochondrial large ribosomal subunit of yeast |
| YEL050C | RML2 | Very sick | Large subunit of Mitochondrial ribosome, similar to *E. coli* L2 ribosomal protein |
| YEL054C | RPL12A | Sick | Large (60S) ribosomal subunit which is similar to Rpl12Bp; rpl12a rpl12b, *E. coli* L11 and rat L12 ribosomal proteins |
| YER117W | RPL23B | Sick | Large (60S) ribosomal subunit, similar to Rpl23Ap, *E. coli* L14 and rat L23 ribosomal proteins |
| YFL034C-A | RPL22B YFL035C-B | Sick | Large (60S) ribosomal subunit, identical to Rpl22Ap and rat L22 ribosomal protein |
| YGR118W | RPS23A | Sick | Ribosomal protein 28 (rp28) of the small (40S) ribosomal subunit involved in translational accuracy; and similar to *E. coli* S12 and rat S23 ribosomal proteins |
| YGR148C | RPL24B RPL30B | Moderate | Protein component of the large (60S) ribosomal subunit, nearly identical to Rpl24Ap and similar to rat L24 |
| YHL033C | RPL8A | Very sick | Protein component of the large (60S) ribosomal subunit, similar to rat L7a ribosomal protein |
| YIL052C | RPL34B | Sick | Protein component of the large (60S) ribosomal subunit, similar to rat L34 ribosomal protein |
| YMR143W | RPS16A | Very sick | Protein component of the small (40S) ribosomal subunit; involved in translation |
| YNL067W | RPL9B | Sick | Large (60S) ribosomal subunit component, similar to *E. coli* L6 and rat L9 ribosomal proteins |
| YOL121C | RPS19A | Sick | Protein component of the small (40S) ribosomal subunit, involved in small ribosomal subunit biogenesis and organization |
| YPL090C | RPS6A | Very sick | Component of Small (40S) ribosomal subunit; involved in translation |
| YPR043W | RPL43A | Very sick | Large (60S) ribosomal subunit, similar to rat L37a ribosomal protein; involved in translation |
| Amino Acids and Protein Production | | | |
| YDL040C | NAT1 AAA1 | Moderate | Involved in N-terminal protein amino acid acetylation, which affects different cellular processes such as the cell cycle, heat-shock resistance, mating, sporulation, and telomeric silencing |
| YER091C | MET6 | Sick | Involved in amino acid biosynthesis; similar to bacterial metE homologs |
| YGR285C | ZUO1 | Moderate | Involved in protein folding, translation fidelity. |
| YLR451W | LEU3 | Sick | Regulate transcription from RNA polymerase II promoter that regulates branched chain amino acid biosynthesis |
| YMR020W | FMS1 | Moderate | Involved in the modification of translation factor eIF-5A; pantothenic acid biosynthesis |
| YOR184W | SER1 ADE9 | Sick | Regulate serine and glycine biosynthesis and the general control of amino acid biosynthesis mediated by Gcn4p |
| YOR303W | CPA1 | Very sick | Small subunit of arginine specific carbamoyl phosphate synthetase, which is involved in the ariginine biosynthetic pathway |
| **rRNA Synthesis** | | | |
| YBL025W | RRN10 | Sick | Protein involved in promoting high level transcription of rDNA and RNA polymerase I promoter transcription factor activity |
| YOL041C | NOP12 | Moderate | Nucleolar protein involved in pre-25S rRNA processing, RNA binding. It is similar to Nop13p, Nsr1p |
