# Supplementary material for: Chemical-genetic profile analysis in yeast suggests that a previously uncharacterized open reading frame, YBR261C, affects protein synthesis
Source: BMC Genomics. 2008 Dec 3;9:583. doi: 10.1186/1471-2164-9-583 (PMC2613417; doi:10.1186/1471-2164-9-583)
Supplement: Additional file 4 — Supplemental Table 4. Descriptions of translation related genes that are phenotypically suppressed by overexpression of TAE1, against treatment with neomycin and/or streptomycin. [file 1471-2164-9-583-S4.doc]

Supplemental Table 4: Descriptions of translation related genes that are phenotypically suppressed by overexpression of *TAE1,* against treatment with neomycin and/or streptomycin.

| Deletion strains which are hypersensitive to: | | Recovery | | Description and Cellular Function |
| --- | --- | --- | --- | --- |
| Neomycin | Streptomycin | Neomycin | Streptomycin |
| Ribosomal Proteins | | | | |
| YBL027W |  | Complete |  | Large (60S) ribosomal subunit component, involved in translation. Similar to rat L19 ribosomal protein; rpl19a and rpl19b |
| YBR189W | YBR189W | Partial | Partial | Small (40S) ribosomal subunit component; Similar to *E. coli* S4 and rat S9 ribosomal proteins; involved in translation and regulate translation fidelity |
| YDL061C |  | Complete |  | Cytosolic small (40S) ribosomal subunit involved in translation; nearly identical to Rps29Ap and similar to rat S29 and *E. coli* S14 ribosomal proteins. Also known as: YS29B |
| YDL075W | YDL075W | Complete | Complete | Ribosomal protein of the large (60S) subunit, similar to rat L31 |
| YDR382W | YDR382W | Partial | Partial | Ribosomal stalk protein involved in the interaction between ribosome and translational elongation factors |
| YGR214W |  | Complete |  | Component of the small (40S) ribosomal subunit, involved in translation; assembly and maintenance of small ribosomal subunit |
|  | YHL033C |  | Partial | Large (60S) ribosomal protein subunit, similar to rat L7 |
|  | YHR010W |  | Complete | Large (60S) ribosomal subunit component, involved in translation |
| YJL080C |  | Partial |  | RNA-binding protein, translate ribosomes via multiple KH domains, demonstrates significant sequence homology to vertebrate vigilins |
| YKL156W |  | Complete |  | Small (40S) ribosomal subunit; similar to rat S27 ribosomal protein |
| YKL167C |  | Complete |  | Mitochondrial large ribosomal subunit involved in translation |
| YKR057W |  | Partial |  | Small (40S) ribosomal subunit protein component involved in translation and telomere maintenance |
| YLR185W |  | Partial |  | Cytosolic protein component of the large (60S) ribosomal subunit, similar to Rpl37Bp and to rat L37 |
| YML024W |  | Partial |  | Ribosomal protein 51 (rp51) of the small (40s) subunit; nearly identical to Rps17Bp and has similarity to rat S17 ribosomal protein |
| YML063W | YML063W | Partial | Complete | Small (40S) ribosomal protein subunit involved in translation; maintenance and assembly of small ribosomal subunit |
|  | YMR143W |  | Partial | Small (40S) ribosomal subunit component; identical to Rps16Bp and similar to *E. coli* S9 and rat S16 ribosomal proteins |
| YMR242C |  | Partial |  | Protein component of the large (60S) ribosomal subunit which is nearly identical to Rpl20Bp and comparable to rat L18a ribosomal protein |
| Translation Control | | | | |
|  | YBL013W |  | Complete | Formyl-Met-tRNA formyltransferase, involved in translation initiation |
| YJL124C |  | Partial |  | Lsm (Like Sm) protein; involved in degradation of cytoplasmic mRNAs, RNA binding |
| YJL209W |  | Complete |  | Mitochondrial protein which interacts with the 5'-UTR of mRNA and has a role mRNA catabolic process |
|  | YKL204W |  | Partial | eIF4E-associated protein, involved in the negative regulation of translation and inhibits cap-dependent translation |
| YMR116C | YMR116C | Complete | Complete | G-beta protein involved in the negative regulation of translation, transposition; ortholog of mammalian RACK1 |
| YNR052C |  | Complete |  | Involved in RNA elongation and regulation of transcription from RNA polymerase II promoter |
|  | YPL178W |  | Complete | Small subunit of the heterodimeric cap binding complex involved in RNA cap binding, mRNA splicing |
| YPR042C |  | Complete |  | PUF protein family member involved in mRNA binding |
| Others | | | | |
| YAL026C | YAL026C | Partial | Complete | Aminophospholipid translocase (flippase) involved in the maintenance of lipid asymmetry; ribosomal small subunit assemble and maintenance |
| YCL009C |  | Complete |  | Subunit of acetolactate synthase which involves in the biosynthesis of branched-chain amino acid |
| YGR159C |  | Complete |  | Nucleolar protein involved in pre-rRNA processing and ribosome biogenesis |
